# Co-Expression Network Analysis Revealed That the *ATP5G1* Gene Is Associated With Major Depressive Disorder

**DOI:** 10.3389/fgene.2019.00703

**Published:** 2019-08-02

**Authors:** Duan Zeng, Shen He, Changlin Ma, Yi Wen, Ying Xie, Nan Zhao, Xirong Sun, Dongxiang Wang, Yifeng Shen, Yimin Yu, Huafang Li

**Affiliations:** ^1^Department of Psychiatry, Shanghai Mental Health Center, Shanghai Jiao Tong University School of Medicine, Shanghai, China; ^2^Department of Psychiatry,Shanghai Jiading District Mental Health Center, Shanghai, China; ^3^Department of Pharmacology and Chemical Biology, Faculty of Basic Medicine, Shanghai Jiao Tong University School of Medicine, Shanghai, China; ^4^Department of Psychiatry, Shanghai Pudong New Area Mental Health Center, Tongji University School of Medicine, Shanghai, China; ^5^Shanghai Key Laboratory of Psychotic Disorders, Shanghai, China; ^6^Clinical Research Center, Shanghai Jiao Tong University School of Medicine, Shanghai, China

**Keywords:** weighted gene co-expression network analysis, major depressive disorder, *ATP5G1*, oxidative stress, biomarker

## Abstract

Major depressive disorder (MDD) is a leading cause of disability worldwide, although its etiology and mechanism remain unknown. The aim of our study was to identify hub genes associated with MDD and to illustrate the underlying mechanisms. A weighted gene co-expression network analysis (WGCNA) was performed to identify significant gene modules and hub genes associated with MDD in peripheral blood mononuclear cells (PBMCs) (*n* = 45). In the blue module (*R* ^2^ = 0.95), five common hub genes in both co-expression network and protein–protein interaction (PPI) network were regarded as “real” hub genes. In another independent dataset, GSE52790, four genes were still significantly down-regulated in PBMCs from MDD patients compared with the controls. Furthermore, these four genes were validated by quantitative real-time polymerase chain reaction (qRT-PCR) in PBMCs from 33 MDD patients and 41 healthy controls. The qRT-PCR analysis showed that ATP synthase membrane subunit c locus 1 (*ATP5G1*) was significantly down-regulated in samples from MDD patients than in control samples (*t* = −2.89, *p*-value = 0.005). Moreover, this gene was significantly differentially expressed between patients and controls in the prefrontal cortex (*z* = −2.83, *p*-value = 0.005). Highly significant differentially methylated positions were identified in the Brodmann area 25 (BA25), with probes in the *ATP5G1* gene being significantly associated with MDD: cg25495775 (*t* = 2.82, *p*-value = 0.008), cg25856120 (*t* = −2.23, *p*-value = 0.033), and cg23708347 (*t* = −2.24, *p*-value = 0.032). These findings indicate that the *ATP5G1* gene is associated with the pathogenesis of MDD and that it could serve as a peripheral biomarker for MDD.

## Introduction

Major depressive disorder (MDD), a common mental disorder with a lifetime prevalence of around 15% in the general population (Tsai, 2006), is predicted to become the second leading cause of disability-adjusted life years in 2020 (Murray and Lopez, 1997). Many different studies have proposed hypotheses regarding the pathogenesis of depression, such as the dysfunctional monoamine theory of depression (Farvolden et al., 2003), the hypothesis of disturbed neuroplasticity (Christoffel et al., 2011), and the inflammatory, oxidative, and nitrosative stress (O&NS) theory of depression (Leonard and Maes, 2012). However, there is no definitive evidence to support any of these theories.

Some evidence has demonstrated that transcriptional alterations in peripheral blood mononuclear cells (PBMCs) may reflect the molecular changes in the brain (Fisar and Raboch, 2008; Fan et al., 2015). Rollins et al. also found that PBMCs and the brain may share a common mRNA expression pattern (Rollins et al., 2010). Moreover, the central nervous system may affect the gene expression of peripheral lymphocytes *via* neurotransmitters, cytokines, or hormones, which may explain the comparable gene expression levels between peripheral blood and some brain tissues (Gladkevich et al., 2004). In addition, several studies have found that the aberrant expression of mRNAs in PBMC samples might be involved in the pathogenesis of MDD (Rocc et al., 2002; Lee and Kim, 2010). Therefore, PBMCs were the focus of our study.

With the development of high-throughput microarray technology, gene expression profiles have been used to identify genes and pathways associated with the pathogenesis of MDD, which have helped to partially illustrate the underlying mechanisms. For example, Tochigi et al. conducted a DNA microarray analysis of major depression using postmortem brains and found 99 differentially expressed genes (DEGs) (Tochigi et al., 2008). A microarray mRNA expression analysis revealed local inflammatory, apoptotic, and oxidative stress in MDD (Shelton et al., 2011). However, most of these studies emphasized only on screening DEGs rather than determining the connection between them, in which genes with similar expression patterns may be functionally related (Tavazoie et al., 1999). Weighted gene co-expression network analysis (WGCNA) is a powerful method for constructing free-scale gene co-expression networks to explore the relationship between different gene sets and clinical features (Langfelder and Horvath, 2008). Thus, to improve the understanding of the biological mechanisms underlying MDD, WGCNA was performed to construct a co-expression network of the relationships between genes and identify significant gene modules and hub genes associated with MDD.

## Materials and Methods

### Study Design and Data Collection

**Figure 1:**
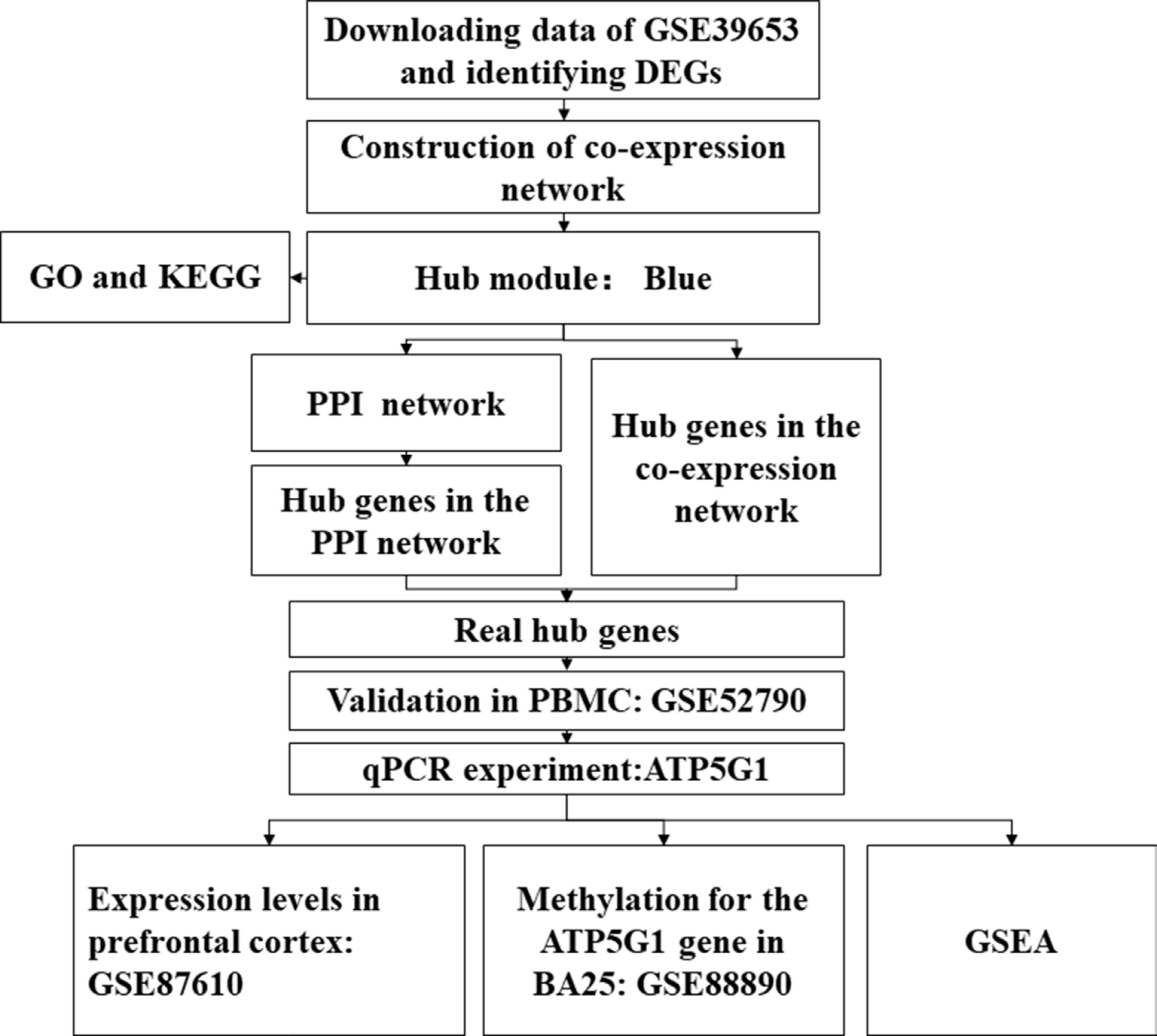
Flow diagram of the data processing, analysis, and validation procedures performed in this study. DEGs, differentially expressed genes; PPI, protein–protein interaction; PBMCs, peripheral blood mononuclear cells; qPCR, quantitative real-time polymerase chain reaction; BA25, Brodmann area 25.

The study design is illustrated in **Figure 1**. First, the normalized gene expression data and relevant clinical information were downloaded from the Gene Expression Omnibus (GEO) database (http://www.ncbi.nlm.nih.gov/geo/). The GSE39653 dataset was used as a training set to screen DEGs, construct co-expression networks, and identify significant modules and hub genes from PBMCs in our study. This dataset, based on the microarray platform of the Illumina HumanHT-12 V4.0 expression beadchip (GPL10558), included 24 adult healthy controls and 21 adult MDD patients (Savitz et al., 2013).

### Data Preprocessing and Screening of DEGs

A microarray annotation file was used to match probes with corresponding genes. For multiple probes corresponding to one gene, the average expression value was calculated as the gene expression value. The limma package in R (Ritchie et al., 2015) was utilized to identify the DEGs between MDD and control samples in the expression data of GSE39653. Genes with *p* < 0.1 were selected for subsequent analyses.

### Co-Expression Network Construction and Identification of Clinically Significant Modules

The “WGCNA” package in R was used to construct a gene co-expression network for the DEGs (Horvath and Dong, 2008; Mason et al., 2009). First, Pearson’s correlation coefficients were calculated for all pairs of genes. Then, a weighted adjacency matrix was constructed using a power function: *a_ij_* = |cor(*x_i_*, *x_j_*)|^β^ (cor(*x_i_*, *x_j_*) = Pearson’s correlation between gene *i* and gene *j*; *a_ij_* = adjacency between gene *i* and gene *j*). β was a soft-thresholding parameter that could emphasize high correlations and weaken low correlations. Next, the adjacency matrix was transformed into a topological overlap matrix (TOM) to measure the network connectivity of genes for network generation (Yip and Horvath, 2007). Finally, with a minimum size (gene group) of 20 for the resulting dendrogram, average linkage hierarchical clustering was performed according to the TOM-based dissimilarity measure to classify genes with similar expression profiles into gene modules (Ravasz et al., 2002).

Module eigengenes (MEs) were regarded as the major component for each gene module in the principal component analysis, which was used to summarize the expression patterns of all genes into a single characteristic expression profile within a given module. Then, the correlation was calculated between MEs and the disease status (MDD vs. control) to identify the relevant modules.

### Functional and Pathway Enrichment Analysis and Protein–Protein Interaction (PPI) Network Construction

Genes in a hub module were uploaded to DAVID Bioinformatics Tool (https://david.ncifcrf.gov/, version 6.7) (Huang et al., 2009a; Huang et al., 2009b) to screen the enriched Gene Ontology (GO) terms and Kyoto Encyclopedia of Genes and Genomes (KEGG) pathways. *p*-value < 0.05 was considered to be significant enrichment.

The PPI network of the genes in the selected module was constructed according to the STRING database (http://www.string-db.org/) and visualized using Cytoscape 3.6.0 (Shannon et al., 2003; Szklarczyk et al., 2017).

### Hub Gene Analysis

Hub genes have high connectivity within a gene module and have been shown to be functionally significant (Goh et al., 2007). In our study, after an interesting module was chosen, hub genes were defined by module connectivity (cor.geneModuleMembership > 0.8) and clinical trait relationship (cor.geneTraitSignificance > 0.2) (Horvath and Dong, 2008; Presson et al., 2008). In the PPI network, genes with a combined score of ≥0.9 and a connectivity degree of ≥10 were also defined as hub genes. The shared hub genes in both the co-expression network and PPI network were regarded as “real” hub genes for further analyses. A Venn diagram (http://bioinformatics.psb.ugent.be/webtools/Venn/) was constructed to identify these “real” hub genes.

To confirm that the real hub genes identified in the GSE39653 dataset were associated with MDD, another independent dataset, GSE52790, based on PBMCs was also downloaded from the GEO database and used as a test set to verify the results of our study. Information about the datasets is provided in **Table 1**.

**Table 1:** Basic information about the datasets used in our study.

Series	Sample size		Tissue type	Type
	MDD	Control		
GSE39653	21	24	PBMCs	Gene expression
GSE52790	10	12	PBMCs	
GSE87610	19	19	L3 pyramidal neurons in the dorsolateral prefrontal cortex	
GSE88890	20	20	Cortical brain region (BA25)	Methylation levels

MDD, major depressive disorder; PBMCs, peripheral blood mononuclear cells; BA25, Brodmann area 25.

### Quantitative Real-Time PCR (qPCR) Experiment

TaqMan qPCR was used to validate the expression changes observed in the four hub genes in 33 MDD patients and 41 age- and sex-matched controls. The ethics committee of Shanghai Mental Health Center approved the research protocol, and all participants provided written informed consent.

Total RNA was extracted from PBMCs using TRIzol reagent (Invitrogen, Carlsbad, California, USA). RNA was converted to cDNA using a PrimeScript RT reagent Kit with gDNA Eraser (Perfect Real Time) (Takara, Kyoto, Japan) according to the manufacturer’s instructions. The TaqMan^®^ Gene Expression Assay was then used to detect the gene expression of the following four genes: *VHL* (Hs03046964_s1), *ATP5G1* (Hs00829069_s1), *COX4I1* (Hs00971639_m1), and *DDOST* (Hs00193263_m1). *18S* (Hs99999901_s1) and β-actin (Hs99999903_m1) were used as reference genes. The 20 μl of PCR reaction mixture contained 1 μl of cDNA, 1 μl of 20× TaqMan assays, 10 μl of 2× TaqMan Universal Master Mix II, No UNG (Applied Biosystems, Foster City, CA, USA), and 8 μl of RNase-free water. The PCR parameters were 50°C for 2 min and 95°C for 10 min, followed by 45 cycles of 95°C for 15 s and 60°C for 1 min. Each sample was run in triplicate in 384-well plates using a LightCycler 480 (Roche) instrument. The relative mRNA abundance was calculated using the 2^−ΔΔCt^ method.

### Expression of the Hub Genes in the Brain and Methylation Analysis

Considering that transcriptional alterations in PBMCs may reflect molecular and cellular changes in the brain (Rollins et al., 2010), the normalized gene expression data in GSE87610 (L3 pyramidal neurons in the dorsolateral prefrontal cortex) (Arion et al., 2017) were downloaded from the GEO database to verify the hub genes that were differentially expressed in the qPCR experiment.

Meanwhile, to detect the methylation levels of the hub genes, the genome-wide patterns of the DNA methylation profiling data (GSE88890) were also downloaded. This dataset includes tissue from the cortical brain region [Brodmann area 25 (BA25)] from 20 depressed MDD suicide completers and 20 non-psychiatric, sudden-death controls (Murphy et al., 2017).

### Gene Set Enrichment Analysis (GSEA)

Twenty-one MDD samples without controls in the GSE39653 dataset were divided into two groups (high-expression and low-expression groups) according to the expression levels of the hub genes. To identify the potential function of the hub genes, GSEA (Subramanian et al., 2005) was performed in our study between the two groups to determine whether a series of pre-defined biological processes were enriched in the gene rank. *p*-value < 0.05 was defined as the cutoff criterion.

### Statistical Analysis

Statistical analysis was carried out using SPSS 20.0 (SPSS Inc., Chicago, IL, USA) and R software. The Shapiro–Wilk test was used to examine the normal distribution of the variables. Depending on the distribution of the data, the independent samples *t* test and the non-parametric Mann–Whitney *U* test were applied to test for differences between groups. *p*-value < 0.05 was considered statistically significant.

## Results

### Differentially Expressed Gene Screening

A total of 3,730 DEGs (1,914 up-regulated and 1,816 down-regulated) below the threshold of *p*-value < 0.1 were selected for subsequent analyses. A volcano plot of the DEGs is shown in **Figure S1**.

### Construction of a Weighted Co-expression Network and Identification of Key Modules

**Figure 2:**
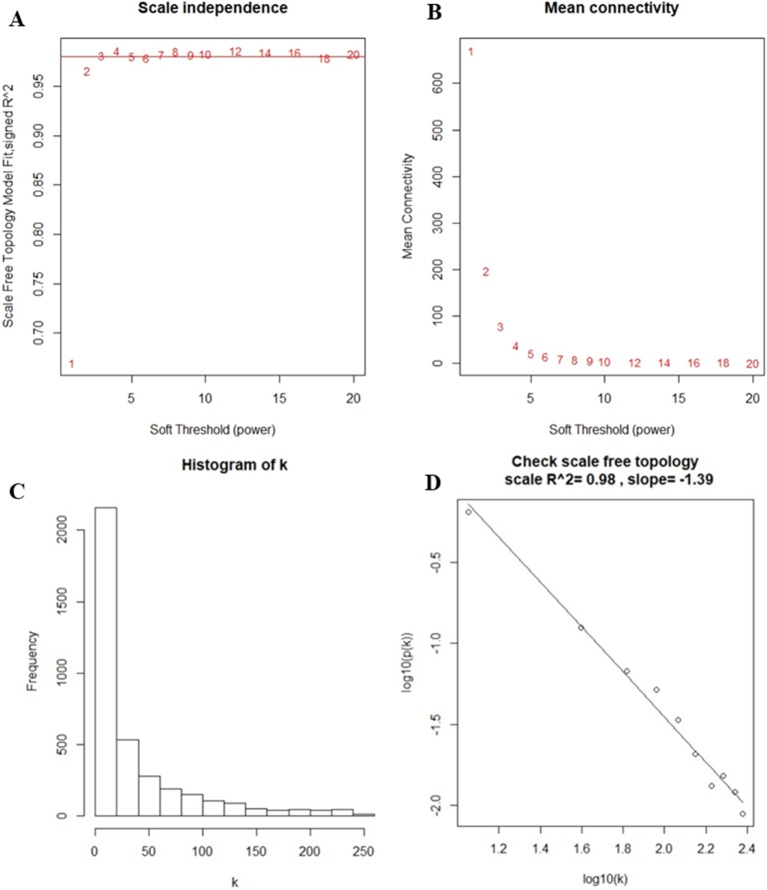
Determination of soft-thresholding power in the weighted gene co-expression network analysis. **(A)** Analysis of the scale-free fit index for different soft-thresholding powers (β). Numbers in the plots represent the corresponding soft-thresholding powers. The approximate scale-free topology can be obtained at a soft-thresholding power of 4. **(B)** Analysis of the mean connectivity for different soft-thresholding powers. **(C)** Histogram of connectivity distribution when β = 4. **(D)** Checking the scale-free topology when β = 4.

**Figure 3:**
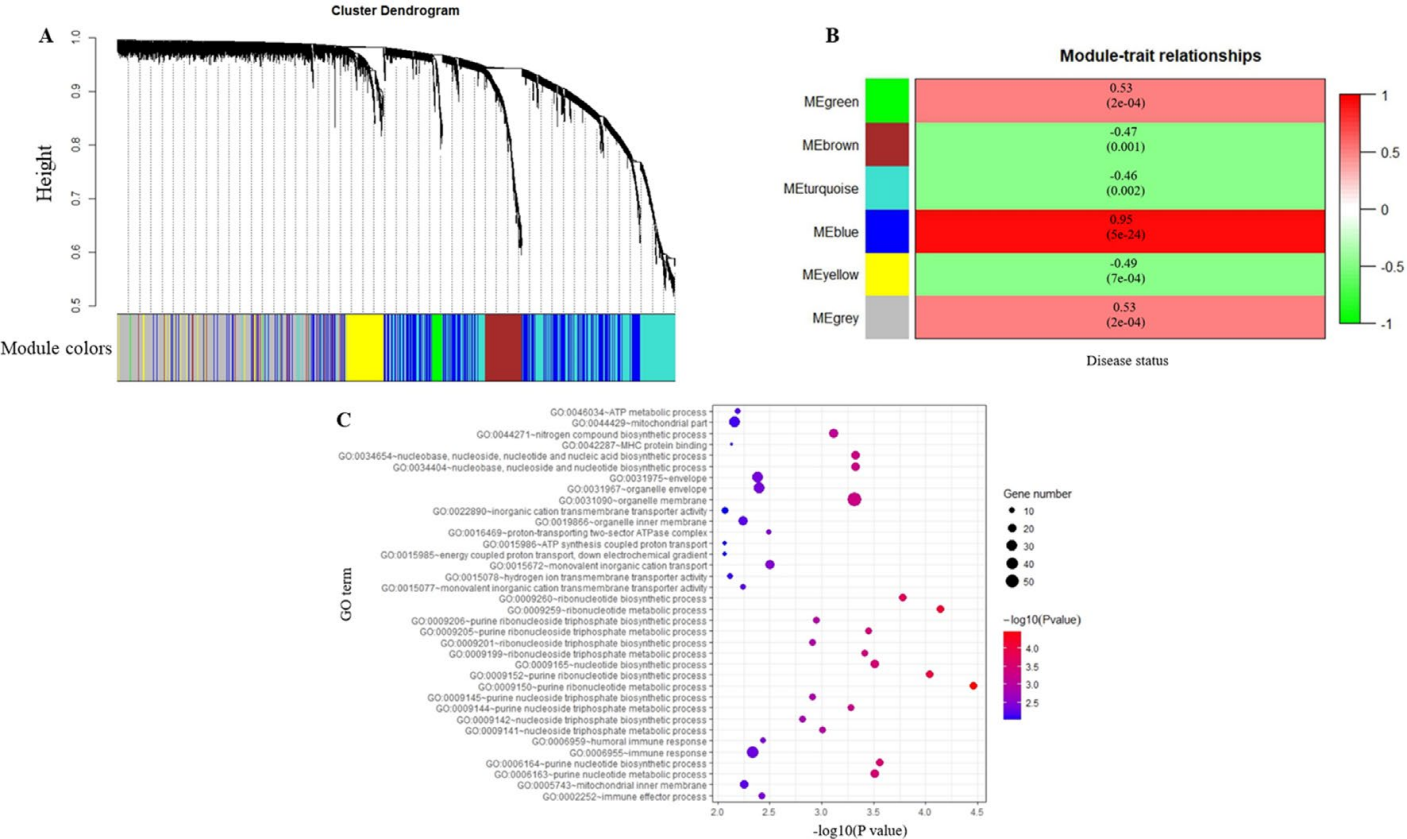
Identification of modules associated with major depressive disorder (MDD) and Gene Ontology analysis. **(A)** Dendrogram of all differentially expressed genes clustered based on a dissimilarity measure (1-TOM). **(B)** Heatmap of the correlation between module eigengenes and the disease status (MDD vs. control). Each cell contains the corresponding correlation and *p*-value. **(C)** Gene Ontology analysis of genes in the blue module.

After the first quality check using the WGCNA R package, no sample was removed from the subsequent analysis in GSE39653 (**Figure S2**). Here, the power of β = 4 (scale-free *R* ^2^ = 0.98) was selected as the soft-thresholding to ensure a scale-free network (**Figure 2**). Then, 3,730 DEGs were divided into different gene modules according to the means of average linkage clustering, and a total of six gene modules (blue, brown, green, grey, turquoise, and yellow) were successfully identified in this study (**Figure 3A**). Furthermore, the correlations were calculated between the different gene modules and the disease status (MDD vs. control) mentioned above. Our results indicated that the module eigengene of the blue module showed the highest correlation among all six gene modules (*R* ^2^ = 0.95, *p*-value = 5e−24) (**Figure 3B**). Thus, we ultimately identified the blue module as the most significant gene module associated with MDD, and the genes in the blue module were extracted for subsequent analyses.

### Functional and Pathway Enrichment Analysis

To obtain further insight into the functions of the genes in the blue module, GO term and KEGG pathway analyses were performed in DAVID database. Most of the GO terms in the blue module were enriched in purine ribonucleotide metabolic and biosynthetic process, ribonucleotide metabolic and biosynthetic process, immune response, immune effector process, and adenosine triphosphate (ATP) synthesis (**Table S1**, **Figure 3C**). KEGG pathway analysis revealed that the genes in the blue module were mainly enriched in oxidative phosphorylation (*p*-value = 0.004). The data indicated that the genes highly associated with MDD were also the most important elements in this module.

### Hub Gene Identification

**Figure 4:**
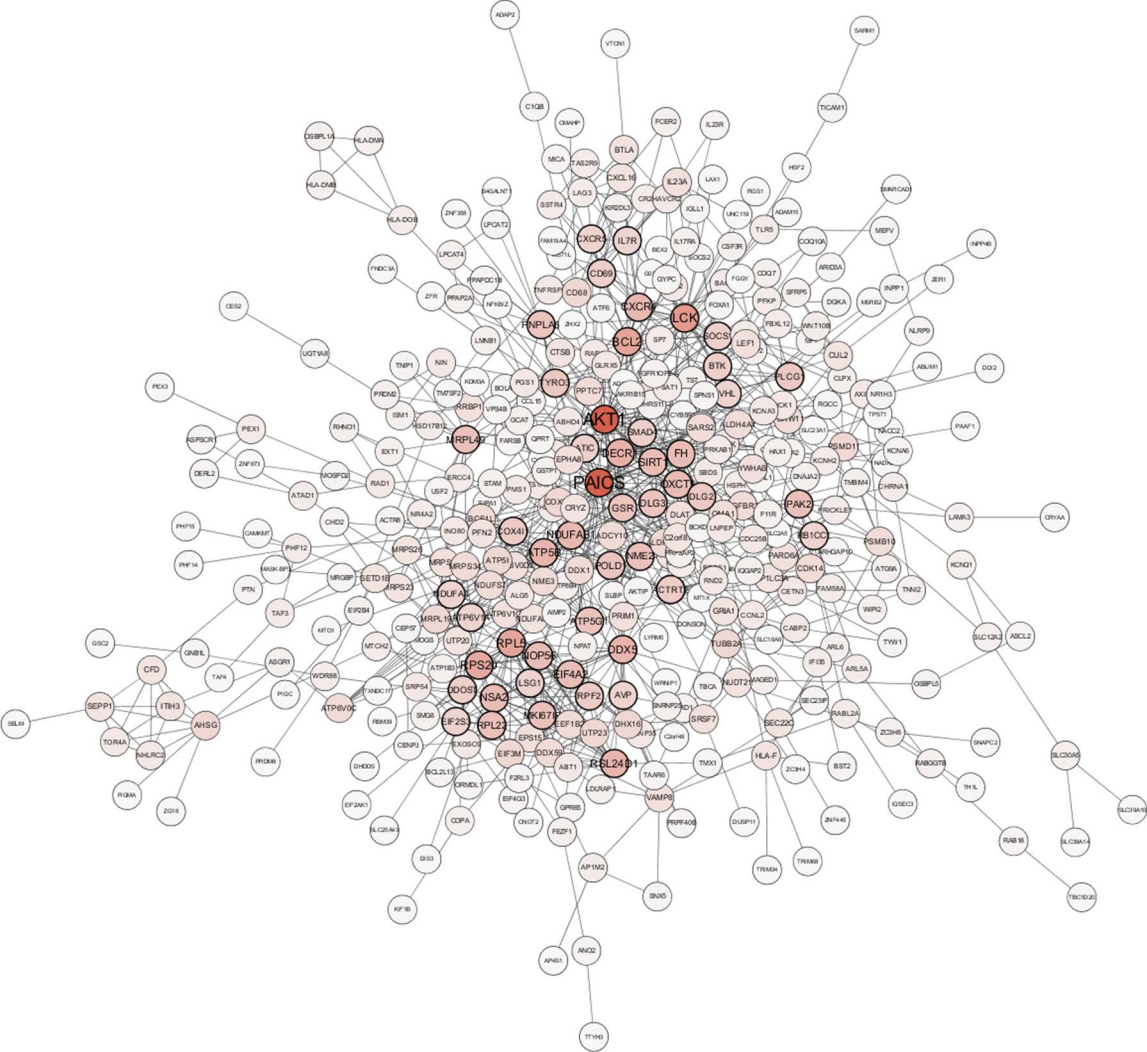
Protein–protein interaction network of genes in the blue module. The color intensity in each node is proportional to the number of edges for the node. The nodes with a bold circle indicate hub genes identified by the PPI network.

**Figure 5:**
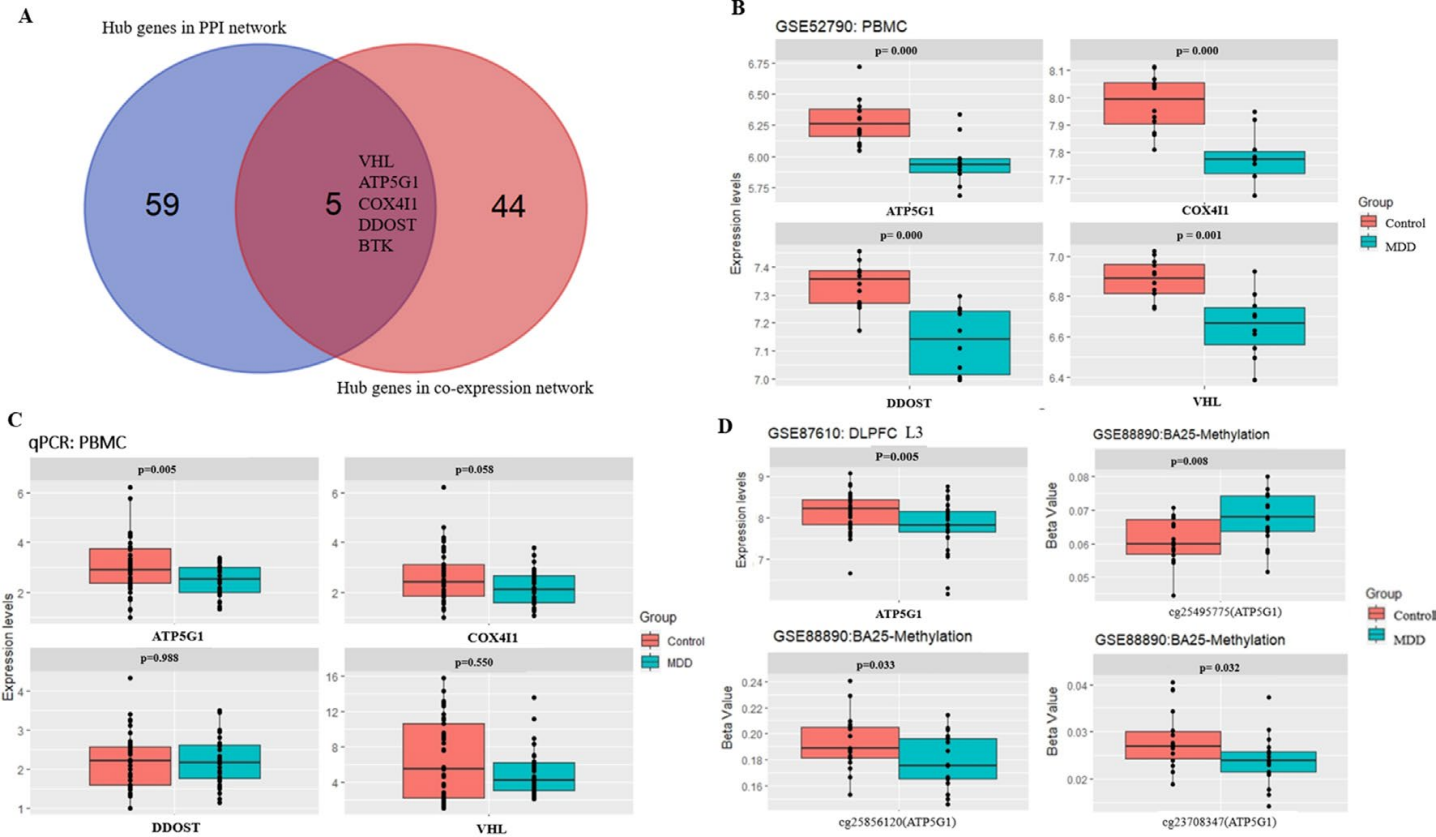
Hub gene detection and validation. **(A)** Selection of real hub genes in the PPI network and co-expression network. **(B)** The expression levels of the four genes (*VHL*, *ATP5G1*, *COX4I1*, and *DDOST*) were correlated with MDD (based on microarray data of GSE52790). **(C)** Relative expression levels of the four genes (*VHL*, *ATP5G1*, *COX4I1*, and *DDOST*) determined by real-time PCR. ATP5G1 was significantly down-regulated in samples from MDD patients compared with the control samples (*p* = 0.005). PCR, polymerase chain reaction. **(D)** The expression levels of *ATP5G1* in L3 pyramidal neurons in the dorsolateral prefrontal cortex (DLPFC) (based on microarray data of GSE87610) and the methylation levels in BA25 (based on microarray data of GSE88890).

A total of 64 genes that were highly connected to the blue module (MM > 0.8 and GS > 0.2) were identified as candidate hub genes (**Table S2**). Moreover, a PPI network was constructed for all genes in the blue module using Cytoscape according to the STRING database, and 49 genes in this PPI network were also taken as hub nodes (**Figure 4**). The five common hub genes (Von Hippel-Lindau tumor suppressor, *VHL*; ATP synthase membrane subunit c locus 1, *ATP5G1*; cytochrome *c* oxidase subunit 4I1, *COX4I1*; dolichyl-diphosphooligosaccharide-protein glycosyltransferase non-catalytic subunit, *DDOST*; and Bruton tyrosine kinase, *BTK*) between the co-expression network and PPI network were screened as “real” hub genes (**Figure 5A**, **Table S2**).

The five shared hub genes were chosen as the candidate genes for further validation. In the GSE52790 dataset, four genes were still significantly down-regulated in the MDD group compared with the control group (*ATP5G1*: *t* = −3.94, *p*-value = 0.0009; *COX4I1*: *t* = −4.66, *p*-value = 0.0002; *DDOST*: *t* = −4.52, *p*-value = 0.0004; *VHL*: *t* = −3.96, *p*-value = 0.0014), whereas *BTK* was not (*p*-value > 0.05) (**Figure 5B**).

### qPCR Validation of the Four Hub Genes

To verify the main conclusion drawn from the microarray results, the relative expression levels of the four hub genes (*VHL*, *ATP5G1*, *COX4I1*, and *DDOST*) in PBMCs were determined using real-time PCR. The clinical characteristics of the participants and the expression levels of the four genes determined by qPCR are shown in **Table 2**. No significant difference in age was detected between MDD patients and healthy controls (38.48 ± 14.92 vs. 33.98 ± 11.60, *t* = 1.443, *p*-value = 0.153). In addition, there was no significant difference in gender distribution between MDD patients (15 males and 18 females) and healthy controls (15 males and 26 females) (χ^2^ = 0.597, *p*-value = 0.482). The RT-qPCR analysis results indicated that *ATP5G1* was significantly down-regulated in samples from MDD patients compared with the control samples (*t* = −2.89, *p*-value = 0.005). However, there were no significant differences for the *VHL*, *COX4I1*, and *DDOST* genes between patients and controls (**Table 2**, **Figure 5C**).

**Table 2:** Demographic and clinical characteristics of the participants in the qPCR experiment.

	MDD (*n* = 33)	Controls (*n* = 41)	*χ*2 or *t* or *z*	*p*-value
Sex (M/F)	15/18	15/26	0.597	0.482^a^
Age (years)	38.48 ± 14.92	33.98 ± 11.60	1.443	0.153^b^
BMI	22.25 ± 3.55	22.43 ± 2.94	−0.231	0.818^b^
Duration of current episode (months)	33.43 ± 60.29			
Duration of illness (months)	77.94 ± 83.60			
Baseline HAMD-17 scores (mean ± SD)	16.12 ± 6.34			
The expression level of *ATP5G1* (mean ± SD)	2.47 ± 0.63	3.06 ± 1.10	**−2.89**	**0.005** ^b^
The expression level of *COX4I1* (mean ± SD)	2.17 ± 0.67	2.65 ± 1.07	−1.90	0.058^c^
The expression level of *DDOST* (mean ± SD)	2.19 ± 0.62	2.19 ± 0.71	0.02	0.988^b^
The expression level of *VHL* (mean ± SD)	5.08 ± 2.73	6.59 ± 4.52	−0.60	0.550^c^

Bold number represents significant p-value.

qPCR, quantitative real-time polymerase chain reaction; MDD, major depressive disorder; BMI, body mass index; HAMD-17, 17-Item Hamilton Rating Scale for Depression; SD, standard deviation.

^a^Chi-square test; ^b^independent samples t test; ^c^Mann–Whitney test.

### Expression of the *ATP5G1* Gene in the Brain and Methylation Analysis

Considering that transcripts in PBMCs could reflect a portion of those expressed in brain tissues (Rollins et al., 2010), the *ATP5G1* expression levels were also evaluated in the prefrontal cortex. When compared with those of the controls, the expression levels of *ATP5G1* were down-regulated in L3 pyramidal neurons in the dorsolateral prefrontal cortex (*z* = −2.83, *p*-value = 0.005, **Figure 5D**). Furthermore, the relationship between the methylation levels of the *ATP5G1* gene and MDD susceptibility was also explored using the GSE88890 dataset. In total, data from 35 BA25 (17 MDD and 18 controls) samples passed the quality control metrics and were used for the analysis. Notably, highly significant differentially methylated positions (DMPs) in the *ATP5G1* gene were identified in the BA25: cg25495775 (*t* = 2.82, *p*-value = 0.008), cg25856120 (*t* = −2.23, *p*-value = 0.033), and cg23708347 (*t* = −2.24, *p*-value = 0.032) (**Figure 5D**).

### Gene Set Enrichment Analysis (GSEA)

**Figure 6:**
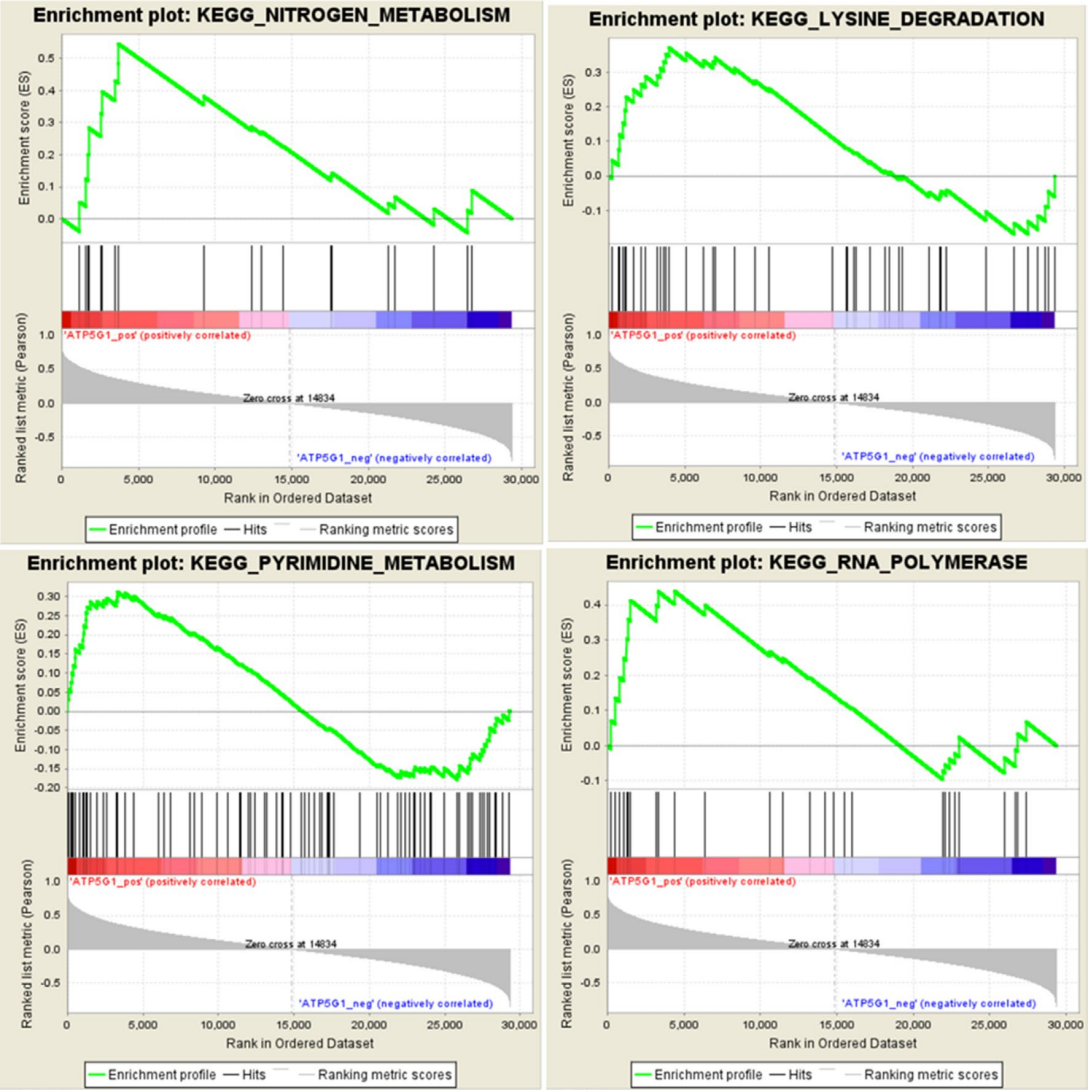
Gene set enrichment analysis (GSEA) for gene sets related to *ATP5G1* expression. The gene sets of “NITROGEN_METABOLISM,” “LYSINE_DEGRADATION,” “PYRIMIDINE_METABOLISM,” and “RNA_POLYMERASE” were enriched in MDD samples in which *ATP5G1* was highly expressed.

To identify potential the function of the hub gene, GSEA was conducted to identify KEGG pathways enriched in the samples in which *ATP5G1* was highly expressed. The gene sets “nitrogen metabolism,” “lysine degradation,” “pyrimidine metabolism,” and “RNA polymerase” were enriched in MDD samples with high *ATP5G1* expression (**Figure 6**).

## Discussion

In this study, WGCNA was performed to identify gene co-expression modules related to the pathogenesis of MDD. After a series of bioinformatics analyses, five hub genes common in both the co-expression network and PPI network were regarded as “real” hub genes. In another independent dataset, GSE52790, four genes (*VHL*, *ATP5G1*, *COX4I1*, and *DDOST*) were still significantly down-regulated in the MDD group compared with the control group. Furthermore, these four genes were validated by qRT-PCR in 33 MDD patients and 41 healthy controls. Overall, the qRT-PCR analysis showed that the *ATP5G1* gene was significantly down-regulated in samples from MDD patients compared with the control samples. Moreover, this gene was significantly differentially expressed in the prefrontal cortex between patients and controls. In addition, highly significant DMPs were identified in the BA25, with probes in the *ATP5G1* gene being significantly associated with MDD. These findings indicate that the *ATP5G1* gene is associated with the pathogenesis of MDD and that it may be a peripheral biomarker for MDD.

*ATP5G1*, a key component of complex V of the oxidative phosphorylation chain, encodes a subunit of mitochondrial ATP synthase and catalyzes ATP synthesis (Vives-Bauza et al., 2010). Natera-Naranjo et al. found that the siRNA-mediated knock-down of axonal *ATP5G1* mRNA resulted in a significant decrease in axonal ATP5G1 protein and ATP levels and an increase in the production of local reactive oxygen species (ROS) (Natera-Naranjo et al., 2012). Other lines of evidence also indicate that *ATP5G1* is involved in the biological process of oxidative phosphorylation and might be associated with oxidative stress (Hu et al., 2009). Many studies have reported that oxidative stress was associated with MDD. Sarandol et al. found that MDD was associated with oxidative stress and that oxidative–antioxidative systems were not affected by 6 weeks of antidepressant treatment (Sarandol et al., 2007). Yanik et al. found that patients with major depression were exposed to oxidative stress and that the degree of oxidative stress could be used to reflect the severity of depression (Yanik et al., 2004). In addition, activated oxidative and nitrosative stress pathways may cause neurodegeneration through different mechanisms such as neuroinflammation and neurotoxic effects (Maes et al., 2011). Thus, we hypothesize that the *ATP5G1* gene is associated with depression by partially influencing oxidative phosphorylation and oxidative stress.

In the present study, we screened the blue module related to MDD using WGCNA. KEGG pathway analysis revealed that the genes in the blue module were enriched mainly in oxidative phosphorylation. Moreover, most GO terms in the blue module were enriched in purine ribonucleotide metabolic and biosynthetic process. Actually, there is evidence that the *ATP5G1* gene is involved in purine metabolism (Reyes et al., 2015). Many studies have showed that purine metabolism, the immune response, and oxidative phosphorylation were associated with MDD (Raedler, 2011; Tobe, 2013; Ali-Sisto et al., 2016). Kaddurah-Daouk et al. found that dysregulated metabolic activity of the purine cycle was associated with several MDD-related systemic responses such as increased pro-inflammatory and oxidative processes (Kaddurah-Daouk et al., 2011; Kaddurah-Daouk et al., 2013). Meanwhile, Niklasson et al. found a correlation between purine metabolite and monoamine metabolite levels in cerebrospinal fluid, which suggested the parallel metabolism of purines and monoamines in the brain (Niklasson et al., 1983). These data suggest that the *ATP5G1* gene may be involved in the pathogenesis of depression by partially influencing purine metabolism.

Moreover, the gene set enrichment analysis revealed that the functions of the *ATP5G1* gene were mainly enriched in terms related to nitrogen metabolism and lysine degradation. Therefore, we hypothesized that this gene was associated with the pathogenesis of MDD by possibly influencing amino acid metabolism. Abnormal amino acid metabolism may induce depression-like behaviors and stress vulnerability (Nagasawa et al., 2012). Ni et al. demonstrated that significant perturbations of metabolites, mainly involving amino acids, played an indispensable role in regulating neural activity in the brain in a rat model of chronic unpredictable mild stress (Ni et al., 2008). Mauri et al. found that plasma and platelet levels of amino acids tended to be higher in depressed patients, especially for lysine, than in healthy controls (Mauri et al., 1998). Ji et al. identified nitrogen metabolism as the most significant pathway associated with SSRI therapy outcome in MDD patients, with several metabolites that were significantly associated (Ji et al., 2011). In addition, the gene set of pyrimidine metabolism was also enriched in MDD samples in which *ATP5G1* was highly expressed. Microarray analysis was used to show that pyrimidine metabolism played a key role in the pathophysiology of MDD (Gao et al., 2015). A recent study that integrated metabolomics and proteomics data reported that purine and pyrimidine metabolism pathways were relevant for the antidepressant treatment response (Park et al., 2017). These data indicate that the *ATP5G1* gene may be associated with depression by influencing amino acid and pyrimidine metabolism.

In conclusion, the co-expression network analysis and qPCR validation results indicated that the *ATP5G1* gene was significantly down-regulated in MDD patients compared with the controls. This study provides preliminary evidence that the *ATP5G1* gene is associated with the pathogenesis of MDD and that it may serve as a peripheral biomarker for MDD.

## Ethics Statement

The Institutional Review Board of Shanghai Mental Health Center approved the research protocol, and all participants provided written informed consent.

## Author Contributions

Authors DZ and SH performed the statistical analyses and wrote the manuscript. Authors CM, YW, YX, and YS managed the literature searches and analyses. Authors NZ and XS were responsible for the diagnosis and clinical assessment of the participants. Author DW provided assistance for the laboratory work. Authors HL and YY offered much constructive advice on this study. All authors read and approved the final manuscript.

## Conflict of Interest Statement

The authors declare that the research was conducted in the absence of any commercial or financial relationships that could be construed as a potential conflict of interest.
