# Supplementary material for: Co-Expression Network Analysis Revealed That the ATP5G1 Gene Is Associated With Major Depressive Disorder
Source: Front Genet. 2019 Aug 2;10:703. doi: 10.3389/fgene.2019.00703 (PMC6688554; doi:10.3389/fgene.2019.00703)
Supplement: Supplementary file 1 [file Table_1.docx]

Table S1. Gene ontology analysis of genes in blue module associated with major depressive disorder.

| **Category** | **Term** | **Gene function** | **Count** | **PValue** |
| --- | --- | --- | --- | --- |
| GOTERM_BP_FAT | GO:0009150 | purine ribonucleotide metabolic process | 16 | 3.45E-05 |
| GOTERM_BP_FAT | GO:0009259 | ribonucleotide metabolic process | 16 | 7.20E-05 |
| GOTERM_BP_FAT | GO:0009152 | purine ribonucleotide biosynthetic process | 14 | 9.08E-05 |
| GOTERM_BP_FAT | GO:0009260 | ribonucleotide biosynthetic process | 14 | 1.64E-04 |
| GOTERM_BP_FAT | GO:0006164 | purine nucleotide biosynthetic process | 15 | 2.77E-04 |
| GOTERM_BP_FAT | GO:0006163 | purine nucleotide metabolic process | 17 | 3.11E-04 |
| GOTERM_BP_FAT | GO:0009165 | nucleotide biosynthetic process | 17 | 3.11E-04 |
| GOTERM_BP_FAT | GO:0009205 | purine ribonucleoside triphosphate metabolic process | 13 | 3.57E-04 |
| GOTERM_BP_FAT | GO:0009199 | ribonucleoside triphosphate metabolic process | 13 | 3.86E-04 |
| GOTERM_BP_FAT | GO:0034654 | nucleobase, nucleoside, nucleotide and nucleic acid biosynthetic process | 17 | 4.70E-04 |
| GOTERM_BP_FAT | GO:0034404 | nucleobase, nucleoside and nucleotide biosynthetic process | 17 | 4.70E-04 |
| GOTERM_BP_FAT | GO:0009144 | purine nucleoside triphosphate metabolic process | 13 | 5.24E-04 |
| GOTERM_BP_FAT | GO:0044271 | nitrogen compound biosynthetic process | 23 | 7.62E-04 |
| GOTERM_BP_FAT | GO:0009141 | nucleoside triphosphate metabolic process | 13 | 9.91E-04 |
| GOTERM_BP_FAT | GO:0009206 | purine ribonucleoside triphosphate biosynthetic process | 11 | 0.001134 |
| GOTERM_BP_FAT | GO:0009201 | ribonucleoside triphosphate biosynthetic process | 11 | 0.001226 |
| GOTERM_BP_FAT | GO:0009145 | purine nucleoside triphosphate biosynthetic process | 11 | 0.001226 |
| GOTERM_BP_FAT | GO:0009142 | nucleoside triphosphate biosynthetic process | 11 | 0.001541 |
| GOTERM_BP_FAT | GO:0015672 | monovalent inorganic cation transport | 21 | 0.003104 |
| GOTERM_BP_FAT | GO:0006959 | humoral immune response | 9 | 0.003645 |
| GOTERM_BP_FAT | GO:0002252 | immune effector process | 12 | 0.00377 |
| GOTERM_BP_FAT | GO:0006955 | immune response | 36 | 0.004599 |
| GOTERM_BP_FAT | GO:0046034 | ATP metabolic process | 10 | 0.006392 |
| GOTERM_BP_FAT | GO:0015986 | ATP synthesis coupled proton transport | 6 | 0.008505 |
| GOTERM_BP_FAT | GO:0015985 | energy coupled proton transport, down electrochemical gradient | 6 | 0.008505 |
| GOTERM_CC_FAT | GO:0031090 | organelle membrane | 57 | 4.85E-04 |
| GOTERM_CC_FAT | GO:0016469 | proton-transporting two-sector ATPase complex | 7 | 0.003225 |
| GOTERM_CC_FAT | GO:0031967 | organelle envelope | 34 | 0.003958 |
| GOTERM_CC_FAT | GO:0031975 | envelope | 34 | 0.004105 |
| GOTERM_CC_FAT | GO:0005743 | mitochondrial inner membrane | 20 | 0.00557 |
| GOTERM_CC_FAT | GO:0019866 | organelle inner membrane | 21 | 0.005682 |
| GOTERM_CC_FAT | GO:0044429 | mitochondrial part | 32 | 0.006878 |
| GOTERM_MF_FAT | GO:0015077 | monovalent inorganic cation transmembrane transporter activity | 10 | 0.005675 |
| GOTERM_MF_FAT | GO:0042287 | MHC protein binding | 5 | 0.007388 |
| GOTERM_MF_FAT | GO:0015078 | hydrogen ion transmembrane transporter activity | 9 | 0.007604 |
| GOTERM_MF_FAT | GO:0022890 | inorganic cation transmembrane transporter activity | 12 | 0.008561 |

Table S2. Hub genes in blue module related with major depressive disorder.

| **Gene** | **Probe** | **Co-expression analysis (cor.geneModuleMembership)** | **Hub gene in PPI network** | **DEG analysis** | |
| --- | --- | --- | --- | --- | --- |
|  |  |  |  | **logFC** | **p** |
| BTK | ILMN_1662026 | 0.830581067 | YES | -0.2136105 | 0.0228061 |
| ATP5G1 | ILMN_1676393 | 0.800586801 | YES | -0.1233555 | 0.0241015 |
| VHL | ILMN_1801984 | 0.802157517 | YES | -0.1121144 | 0.0306727 |
| COX4I1 | ILMN_1652207 | 0.912881476 | YES | -0.2026495 | 0.0580843 |
| DDOST | ILMN_1734231 | 0.806162639 | YES | -0.09634 | 0.0901789 |
| YWHAB | ILMN_1694385 | 0.871516639 | NO | -0.1857667 | 0.0018727 |
| MEFV | ILMN_1804738 | 0.84500178 | NO | -0.1831082 | 0.0114115 |
| TMBIM4 | ILMN_1664750 | 0.861015617 | NO | -0.1538987 | 0.0134954 |
| CXCL16 | ILMN_1672278 | 0.812935068 | NO | -0.1562686 | 0.0156529 |
| LOC653888 | ILMN_1804530 | 0.805081998 | NO | -0.2815089 | 0.0179232 |
| TBCA | ILMN_1726239 | 0.888664737 | NO | -0.2159654 | 0.0180911 |
| LOC646531 | ILMN_1669424 | 0.820581675 | NO | -0.2340168 | 0.0200969 |
| SIPA1 | ILMN_1682930 | 0.80403719 | NO | -0.141057 | 0.0215451 |
| XPO7 | ILMN_2174884 | 0.844892009 | NO | -0.1387435 | 0.0265533 |
| TRIM34 | ILMN_1657709 | 0.819403332 | NO | -0.0840529 | 0.026772 |
| LOC100133568 | ILMN_3242535 | 0.855420982 | NO | -0.46512 | 0.0271266 |
| BST2 | ILMN_1723480 | 0.802589653 | NO | -0.224394 | 0.0271926 |
| BRI3P1 | ILMN_3253304 | 0.92568069 | NO | -0.3880872 | 0.0278649 |
| VAMP8 | ILMN_2190084 | 0.804697365 | NO | -0.1869937 | 0.0288134 |
| CYB5R1 | ILMN_1729237 | 0.865649357 | NO | -0.2233965 | 0.0293641 |
| LOC100132394 | ILMN_3249578 | 0.829252633 | NO | -0.3255357 | 0.0296834 |
| CTBP2 | ILMN_1691294 | 0.815884523 | NO | -0.0716156 | 0.0312236 |
| COX5A | ILMN_1704477 | 0.877642341 | NO | -0.2137234 | 0.0316633 |
| TRIM16L | ILMN_2193443 | 0.834028511 | NO | -0.5083938 | 0.0363751 |
| TMEM9B | ILMN_2100815 | 0.851488604 | NO | -0.1745523 | 0.0382264 |
| SLC27A3 | ILMN_1719627 | 0.90270574 | NO | -0.2294394 | 0.0409614 |
| MSRB2 | ILMN_1657977 | 0.871833384 | NO | -0.3171586 | 0.0410009 |
| NINJ2 | ILMN_1731745 | 0.847579812 | NO | -0.2279633 | 0.0425833 |
| ADAP2 | ILMN_1763000 | 0.818211635 | NO | -0.2372213 | 0.0453322 |
| TST | ILMN_1691572 | 0.855992344 | NO | -0.2883356 | 0.0453826 |
| LOC728873 | ILMN_3298582 | 0.864720367 | NO | -0.2187148 | 0.0464446 |
| TMX1 | ILMN_1691181 | 0.807231722 | NO | -0.0889022 | 0.0476143 |
| FGD2 | ILMN_2115005 | 0.864186622 | NO | -0.1608052 | 0.0486902 |
| NDUFS7 | ILMN_1669966 | 0.919077787 | NO | -0.300094 | 0.0495364 |
| SDF2L1 | ILMN_1749213 | 0.862088747 | NO | -0.2230441 | 0.0497057 |
| RARA | ILMN_1659206 | 0.806930835 | NO | -0.0507072 | 0.0561645 |
| CES2 | ILMN_1696675 | 0.866182363 | NO | -0.1892026 | 0.0597059 |
| GSTP1 | ILMN_1679809 | 0.877968228 | NO | -0.2104361 | 0.0599483 |
| TBCCD1 | ILMN_1796411 | 0.864274253 | NO | -0.1293461 | 0.0605902 |
| LOC100134172 | ILMN_3244405 | 0.867440515 | NO | -0.3811129 | 0.0615754 |
| SLC16A3 | ILMN_2364022 | 0.824341391 | NO | -0.2016458 | 0.0651741 |
| PSMB10 | ILMN_1683026 | 0.913044968 | NO | -0.2335708 | 0.0653841 |
| PLEKHO1 | ILMN_1694213 | 0.833893268 | NO | -0.1328453 | 0.0654786 |
| SLC25A43 | ILMN_1662097 | 0.811408331 | NO | -0.0963484 | 0.0663291 |
| PP8961 | ILMN_2067421 | 0.808379706 | NO | -0.1747572 | 0.0666497 |
| HPS6 | ILMN_1718537 | 0.818098792 | NO | -0.1791231 | 0.0684726 |
| GLIPR2 | ILMN_1652631 | 0.909630009 | NO | -0.1969453 | 0.068763 |
| NDUFV3 | ILMN_1765500 | 0.800714714 | NO | -0.2061671 | 0.0717609 |
| NDUFAF3 | ILMN_1702120 | 0.824525129 | NO | -0.0977277 | 0.0733198 |
| C17orf71 | ILMN_1807533 | 0.813841945 | NO | -0.112122 | 0.0756006 |
| ATF6 | ILMN_1703471 | 0.848383673 | NO | -0.1391212 | 0.0768203 |
| ABHD8 | ILMN_1712707 | 0.83989928 | NO | -0.1653362 | 0.0773075 |
| LOC100129543 | ILMN_3252446 | 0.874262129 | NO | -0.5139233 | 0.0796525 |
| C20orf20 | ILMN_1790136 | 0.871290342 | NO | -0.1527863 | 0.0800215 |
| LOC100134363 | ILMN_3236122 | 0.876864533 | NO | -0.2701336 | 0.08316 |
| LOC641522 | ILMN_2141030 | 0.882721604 | NO | -0.3319141 | 0.0831768 |
| LOC100130886 | ILMN_3241091 | 0.844946564 | NO | -0.1603846 | 0.083525 |
| JDP2 | ILMN_1747205 | 0.838957119 | NO | -0.1930429 | 0.0864858 |
| FAM131A | ILMN_1729217 | 0.860288246 | NO | -0.1434231 | 0.090073 |
| SNAPC2 | ILMN_1698478 | 0.820918587 | NO | -0.1139094 | 0.0918779 |
| BCKDK | ILMN_1693394 | 0.882686666 | NO | -0.1925197 | 0.09229 |
| CENTA1 | ILMN_2047511 | 0.874435681 | NO | -0.1739033 | 0.0935338 |
| LRP3 | ILMN_2127605 | 0.827164205 | NO | -0.2250344 | 0.0937398 |
| SNHG10 | ILMN_2409078 | 0.85606088 | NO | -0.45091 | 0.0979778 |


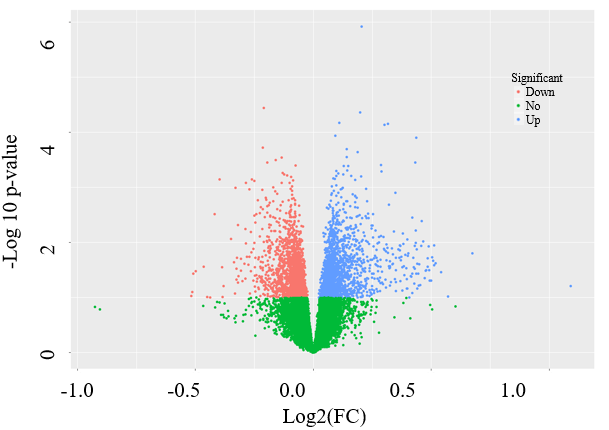


Figure S1. Volcano plots representation of differential expression analyses. The criteria for selection of DEGs was set as P < 0.1.


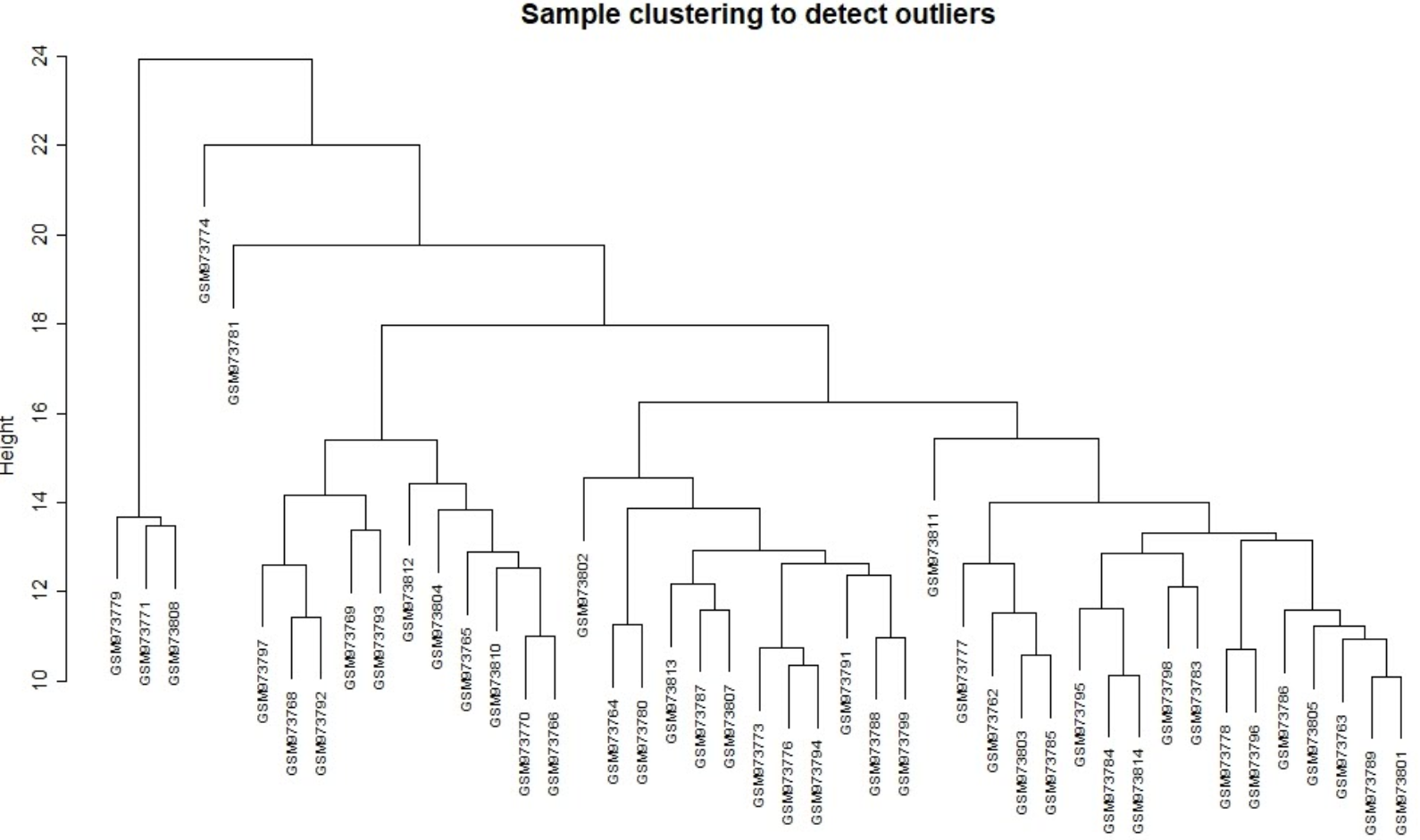


Figure S2. Samples clustering of GSE39653 to detect outliers. No samples were removed from subsequent analysis in the test dataset.
